# Technological Challenges During Video Visits with Veterans for Medication Management

**DOI:** 10.1093/geroni/igaf122.4207

**Published:** 2025-12-31

**Authors:** Michael Zanchelli, Kristal Xie, Chelsea Hawley, Sheikh Hossain, Akanksha Samant, William Hung

**Affiliations:** James J. Peters VAMC, Bronx, New York, United States; University of Hawaii at Manoa, Honolulu, Hawaii, United States; VA Bedford Healthcare System, Bedford, Massachusetts, United States; James J Peter VA Medical Center, New York, New York, United States; James J Peters VA Medical Center, Bronx, NY, New York, United States; James J. Peters Department of Veterans Affairs Medical Center, New York, New York, United States

## Abstract

There is limited understanding of technological barriers that occurs leading up to and during video visits for older adults. Successful implementation of video visit to deliver care requires recognizing the specific barriers faced by older adults and developing targeted strategies to address these factors. We share the barriers faced and how we overcame them in 129 video visits with older Veterans. As part of a randomized controlled trial at Bronx and Bedford VAs, 129 Veterans attempted a video consultation with a clinical pharmacist from their home, to assess their medications and answer medication-related questions. Participants were aged ≥65 years, taking ≥5 daily VA medications, having ≥2 chronic conditions, ≥ 1 VA primary care visit within past year with no history or diagnosis of dementia (MoCA score > 24/30 or telephone MoCA [t-MoCA] score ≥ 18/22). Of 129 Veterans, 120 (93%) successfully completed a video visit, with 95% using their own device and 64% using smartphones. Most participants (79%) connected on their first attempt, although 58% required assistance. Average assistance last 17 minutes (range 1-33), primarily provided by study coordinators (74%), and pharmacists (11%). The most common challenges included connection/bandwidth problems (25%), technology literacy (20%), and hearing difficulties (13%). The most common methods for overcoming these challenges were proactive assessment of health literacy, cognitive status, technology comfort, device ownership, and prior video visit experience, as well as the opportunity to practice a video visit before the actual visit to work out technology kinks.

